# Au L-shell x-ray emission induced by 154.3–423.9 MeV/u C^6+^ ions

**DOI:** 10.1038/s41598-022-23830-5

**Published:** 2022-11-10

**Authors:** Xianming Zhou, Jing Wei, Rui Cheng, Yanning Zhang, Yanhong Chen, Changhui Liang, Xiaoan Zhang, Yongtao Zhao

**Affiliations:** 1 Ion Beam and Optical Physics Joint Laboratory of Xianyang Normal University and IMP, CAS, Xianyang Normal University, Wenlin Rd. 01, Xianyang, 712000 China; 2 Institute of Modern Physics, Chinese Academy of Sciences, Lanzhou, 730000 China; 3 School of Science, Xi’an Jiaotong University, Xi’an, 710049 China

**Keywords:** Atomic and molecular collision processes, Physics

## Abstract

The L-shell x-ray emissions of gold are investigated for the bombardment of high energy C^6+^ ions in the high energy region of 154.3–423.9 MeV/u. Due to the multiple ionization of outer-shell electrons at the movement of L x-ray emission, the blue shift of the experimental x-ray energy and an enhancement of the relative intensity ratios of Lι, Lβ–Lα x rays are observed. Using the improved thin target formula and considering the effect of multiple ionization on atomic parameters, the L-subshell x-ray production cross sections are extracted from the counts and compared with the theoretical estimations of BEA, PWBA and ECPSSR. It is found that the relative corrections of ECPSSR on PWBA can be ignored in the present experimental energy region. The calculations of PWBA and ECPSSR are almost identical and both are larger than the experimental results. The BEA is in better agreement with the experiment as a whole.

## Introduction

High energy ions have been extensively exploited in different ways in various disciplines, such as, astrophysics, atomic physics, plasma physics, materials physics, biomedicine and so on, for both fundamental research and practical application^1–6^. High energy heavy ions beam is also an alternative driver for the indirect-drive inertial confinement fusion^7–10^. Here, the energy of the driving beam is first converted into x-ray radiation by bombarding of the high energy pulsed beam with a hohlraum made of high-Z material, and then the radiation drives the fuel pellet to implode. Detail knowledge of the conversion efficiency of x-ray radiation and the properties of the radiation field is urgently needed to such indirect drive implosion. In addition, highly charged ions widely exist in the cosmic objects and dense plasma associated with warm-dense-matter and high-energy-density-matter, where they interacts with the surrounding particles to produce x-ray emission, which is affected by the surrounding environment such as the electron temperature, density and other plasma parameters. In turn, the measurement of x-ray emission provides a viable method for the diagnosis of dense plasma^11,12^. Therefore, it is of great significance to further study the x-ray emission induced by high energy heavy ions.

The characteristic x-ray emission, the consequential result from the decay of the projectile ion or inner-shell vacancies produced by collision of energetic ion with atom, provides important information to the arrangement of atomic orbital electron and inner shell process in collisions. In the low and intermediate energy region, a great deal of experimental works have been carried out focusing on such process by x-ray measurement^13–18^, and many well-known theories have been developed to describe such inner-shell ionization, such as, binary encounter approximation (BEA)^19^, plane wave Born approximation (PWBA)^20^, modified PWBA model by energy-loss, Coulomb-repulsion, perturbed-stationary-state and relativistic (ECPSSR) and refined ECPSSR at low energies with modification of united and separated atom approximation (ECUSAR)^21–23^. However, only a limited number of studies have been undertaken in the very high energy region, especially for the fast ions with energy greater than one hundred MeV per mass unit (MeV/u)^24–28^. It is unclear which existing theory molded is more suitable in such a high energy region. Further experiments are required to systematically examine the various simulations.

In energetic ion-atom collisions, the ionization of an inner-shell electron may be accompanied by the ionization of another inner or one or more outer-shell electrons in the same atom. This action, called multi-ionization, is related to the atomic number, charge state, incident energy of projectile and the atomic number of the target atom^29,30^. This can be determined by measuring the blue shift of x-ray energy using a low-resolution semiconductor detector, or analyzing the satellite and hyper-satellite of x-ray spectra with a high-resolution crystal spectrometer. Double K shell ionization produced by fast ions with energies in the tens of MeV/u has been extensively studied by observing the K-shell hyper-satellite lines^31–34^. The multiple ionization induced by low-energy light ions has also been reported by comparing the relative intensity ratio of subshell x-rays^35–37^. It is proposed that the same is anticipated for the bombardment of high energy heavy ions with energies in hundreds of MeV/u.

Therefore, in this work, we would like to present the experimental results of the L-shell x-ray emission of Au produced by the bombardment of high energy C^6+^ ions with energies in hundreds of MeV/u. To confirm the multiple ionization by high energy heavy ions, the energy and the relative intensity ratios of the sub-shell x-ray is obtained and compared with the atomic data. The experimental x-ray production cross sections are calculated and compared with various theoretical simulations to test and verify the applicability of the theory.

## Experimental method

The experiment was performed at the cancer therapy terminal at the national laboratory of Heavy Ion Research Facility in Lanzhou (HIRFL) in the Institute of Modern Physics, Chinese Academic of Science (IMP, CAS). The highly charged C^6+^ ions are produced and extracted from the Electron Cyclotron Resonance (ECR) ion source, and then accelerated by the main Cooling Storage Ring (CSRm). The beam quality is improved by a new generation electron cooler, and the corresponding uncertainty of the energies is less than 0.22%. The beam with a duration of 3 ns, and a pulse distance of 15 s was extracted directly into the air and impact perpendicularly on the target after collimation. The beam size measured with a CsI(Tl) crystal is about 5 × 5 mm, and the beam intensity of impacting on the target is about 10^8^ ions/pulse. The initial energies of the accelerated C^6+^ ions in the CSRm are 165, 214, 300, 350 and 430 MeV/u, and the actual energies of the projectile impacting on targets before the collision are about 154.3, 205.0, 292.7, 343.3 and 423.9 MeV/u, respectively, owing to the energy loss passing through the Beryllium window and the air between the target and the exit of the beam line terminal. The number of the incident projectile was measured indirectly by the combined use of a Faraday cup and the counts given by the counter fixed around the exit of the beam line terminal.

**Figure 1:**
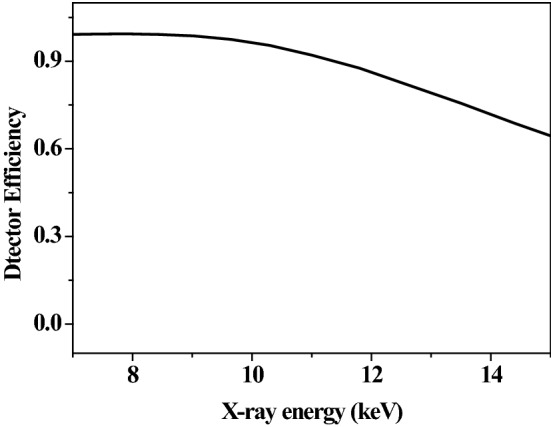
Efficiency of the Silicon Drift Detector.

The x-rays were observed by a silicon drift detector (SDD) with an effective detection area of 7 mm^2^ and a 12.5 μm Beryllium window in the front of the detector. The SDD was placed 100 mm from the target surface and at a 135 degree angle to the beam direction. The solid angle is about 7 × 10^–4^ Sr. The detector has an effective energy range of 0.5–14.5 keV when the gain was selected at 100, and an energy resolution of about 136 eV at 5.9 keV when the peaking time is set at 9.6 μs. The energy calibration is performed using simultaneously the two standard radioactive sources of ^55^Fe and ^241^Am, and then tested by measuring the K-shell x-rays of Al, V and Fe produced by photon irradiation. In this way, an accurate measurement of the x-ray energy can be guaranteed. The SDD intrinsic efficiency, which combines the transmission effect through the Beryllium window and the interaction in the silicon detector, is determined and provided by the manufacturer of Amptek Company, and is shown in Fig. 1. The target of gold (Au) has a purity of 99.999% with surface area of 15 × 20 mm^2^ and a thickness of 0.2 mm in the present work.

## Results and discussion

1. Au L-shell x-ay spectra induced by C^6+^ ions

**Figure 2:**
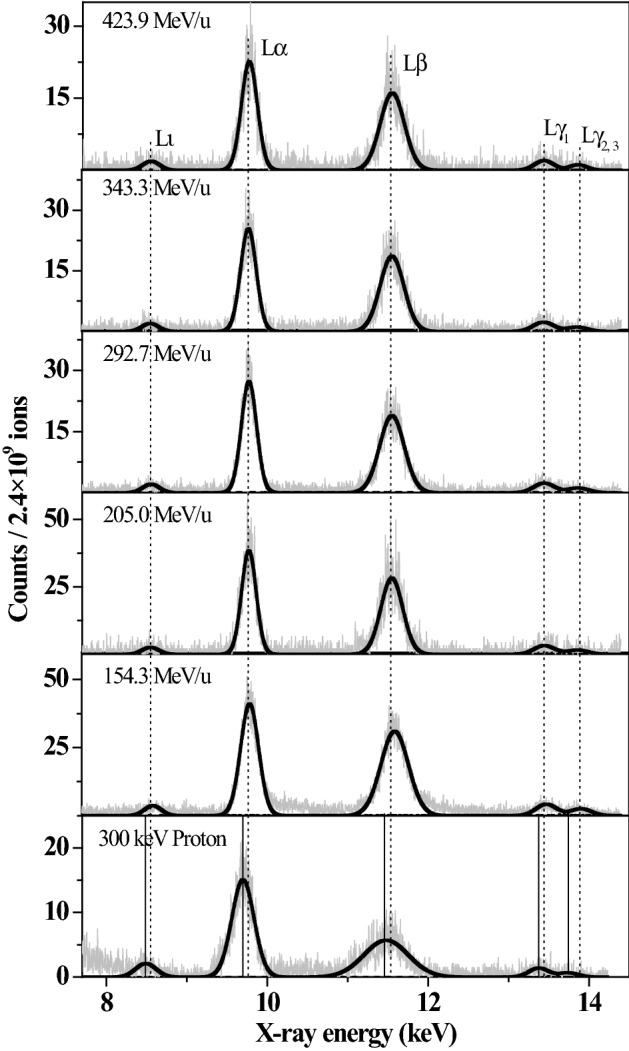
Au L-shell X-ray spectra induced by high energy C^6+^ ions with various incident energy, and compared with that induced by protons.

Figure 2 shows the typical x-ray emission spectra induced by C^6+^ ions with various incident energies in the range of 154–424 MeV/u. As a comparison, the spectrum by the impact of 300 keV protons is also given, which is measured experimentally on the 1# terminal of the 320 kV high voltage experimental platform at the Institute of Modern Physics, Chinese Academy of Sciences (IMP, CAS) in Lanzhou, China. Due to the absorption of air and the low detection efficiency of the detector in the low energy region, the low energy x-ray spectrum is not recorded in this work. The present spectra are normalized by the number of incident ions (it is about 2.4 × 10^9^ ions) and are well fitted by a nonlinear curve Gaussian fitting program of origin software. For example, the goodness of fit (R-square, R^2^) is larger than 0.99. They are regarded as the x rays of Au from the relative decay of L-subshell vacancies.

In Fig. 2, it is obvious that the spectrum consists of four distinct sets of lines, which are identified as Lι, Lα, Lβ and Lγ x ray of Au^38,39^. Lι x ray comes from the radiation transition of M_1_ shell electron to L_3_ shell vacancy. Lα actually comprise of two x rays which cannot be easily discriminated due to the limitation of the detector, Lα_1_ and Lα_2_ x ray, and which result from the transitions of M_5_-L_3_ and M_4_-L_3_, respectively. Lβ mainly contains six distinguishable spectral lines emitting from the radiation transitions of M_4_–L_2_, N_4, 5_–L_3_, M_3_–L_1_, M_2_–L_1_, O_4, 5_–L_3_ and N_1_–L_3_, which are Lβ_1_, Lβ_2 15_, Lβ_3_, Lβ_4_, Lβ_5_, and Lβ_6_ x ray, respectively. Lγ is mainly two group lines, Lγ_1_ and Lγ_2, 3_ x ray, which are the results of transitions of N_4_–L_2_ and N_3, 2_–L_1_, respectively.

One can also find from Fig. 2 that the spectral structures are basically similar for C^6+^ ions with different energies, but they are different from that produced by protons, which can be regarded as a standard spectrum of single ionized atom. Compared to the x-ray emission by protons, the experimental center position of the spectral line induced by the high energy heavy C^6+^ ions is shifted toward the high energy direction. The emission of Lβ x rays are enhanced compared to that of Lα. That can be interpreted from the influence of multiple ionization of outer-shells on the x-ray emission.

2. Energy shift of the x ray result from multiple ionization

Multiple ionization could be produced in high energy ion-atom collisions or by the impact of lower energy light ions, and can be largely enhanced by heavy-ions bombardment^40–44^. This process causes the formation of multiple vacancies in the outer shells. These vacancies may not be filled before the radiative decay of the inner-shell vacancy, and act as spectators at the moment of inner-shell x-ray emission. This result in a reduction in the screening of nuclear charge and then give rise to increase of the binding energy for all energy levels. As a result, the energy of the x ray emitting from multiply ionized ions is blue shifted.

Table 1 displays the measured energies of Au L-subshell x rays, as well as the results of proton which are almost consistent with the theoretical calculations for single ionized atom and can be regarded as atomic data. The data of high energy C^6+^ ions are all larger than the atomic data, and there is no obvious regular change with the increase of incident energy. Only the result at the incident energy of 154.3 MeV/u is slightly larger than those at other energies. For example, when the energy is 154.3 MeV/u, the blue shifts of Lι, Lα, Lβ, Lγ_1_ and Lγ_2, 3_ x ray are 91 ± 5, 83 ± 3, 113 ± 3, 93 ± 5 and 166 ± 7 eV, respectively. However, the average of those shift are 57 ± 4, 69 ± 4, 76 ± 5, 62 ± 6 and 123 ± 8 eV, when the incident energies are at 205.0, 292.7, 343.3 and 423.9 MeV/u, respectively. It is indicated that with the ionization of L-subshell, the M- and N-shell electrons are multiply ionized by the bombardment of high energy C^6+^ ions. In the present experiment, the extent of such multiple ionization is almost constant as a function of the incident energy. This can also be confirmed by the results of the relative intensity ratio of the L-subshell x ray discussed below.

**Table 1:** Au L-subshell x-ray energies induced by high energy C^6+^ ions and 300 keV H^+^, and the atomic data ^38,39^.

	Lι (eV)	Lα (eV)	Lβ (eV)	Lγ_1_ (eV)	Lγ_2, 3_ (eV)
Atomic	8494	9703	11,475	13,379	13,736
Proton	8491 ± 4	9698 ± 3	11,477 ± 5	13,374 ± 4	13,739 ± 5
154.3 MeV/u	8585 ± 5	9786 ± 3	11,588 ± 3	13,472 ± 5	13,902 ± 7
205.0 MeV/u	8549 ± 5	9772 ± 5	11,548 ± 5	13,444 ± 7	13,859 ± 8
292.7 MeV/u	8556 ± 4	9773 ± 3	11,550 ± 4	13,442 ± 5	13,863 ± 6
343.3 MeV/u	8551 ± 3	9771 ± 5	11,554 ± 4	13,437 ± 4	13,852 ± 9
423.9 MeV/u	8549 ± 5	9772 ± 4	11,550 ± 5	13,440 ± 6	13,862 ± 8

In the approximation of independent-particle framework, multiple ionization can be treated as simultaneous independence single ionization of the orbital electrons, taking electrons correlation effects and subsequent excitation by non-radiation transition out of account^3,29,30,45^. Considering the uniformity of ionization probability of each electron in the same shell, the multi-ionization cross section can be represented by the binomial distribution. The multi-ionization degree is positive to the single ionization cross section^40,45^. The high energy ion-atom collision in the sample can be regard as a binary process, where the inner-shell ionization is mainly determined by the Coulomb interaction between the projectile ions and the electrons in the atomic shell. The cross section of single ionization can be estimated by the BEA model.

According to the calculation of BEA^19^, the ionization cross sections of M and N shells of Au in this experiment are on the order of 10^5^ and 10^6^, respectively, which are 2 and 3 orders of magnitude larger than that of the L shells, respectively, and this cross section decreases with the increase of incident energy. Theoretically, it is expected that the multi-ionization degree of outer-shells is dwindled with increasing incident energy. However, this is not the case. Although, the ionization cross section decreases with increasing incident energy, the extent of such decrease is less than 60%, and it only changes within the same order of magnitude. For instance, the M-shell ionization cross section at the energy of 423.9 MeV/u is only about 40% of that at 154.3 MeV/u. As the incident energy increases from 154.3 MeV/u to 205.0 MeV/u, to 292.7 MeV/u, to 343.3 MeV/u and to 423.9 MeV, the extents of reduction are 35, 15, 12 and 17%, respectively. Therefore, it is proposed that such slow reduction of single ionization cross section does not cause a significant change in the multi-ionization degree in the experiment. This is consistent with the experimental results given in Table 1. To be more specific, the reduced slop of the cross section for the incident energy increasing from 154.3 to 205.0 MeV/u is larger than that in the energy region of 205.0–423.9 MeV/u. When the incident energy is set to 154.3 MeV/u, the cross section of single ionization has a largest value. It is indicated that the multi-ionization degree is at its maximum at the incident energy of 154.3 MeV/u. This is just the reason why the blue shift is the largest at 154.3 MeV/u, as shown in Table 1.

3. Enhancement of the relative intensity ratio of L-subshell x ray caused by multiple ionization

**Figure 3:**
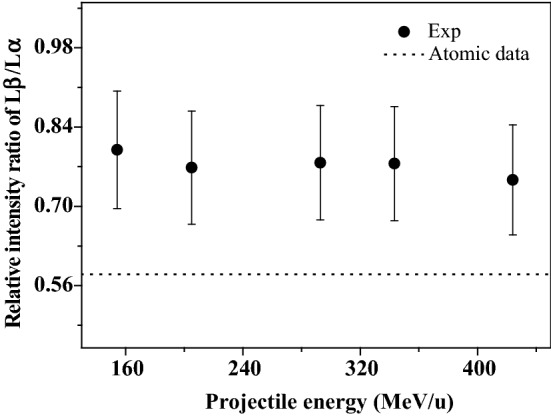
Relative intensity ratios of Au Lβ–Lα x-ray induced by C^6+^ ions at different incident energy.

**Figure 4:**
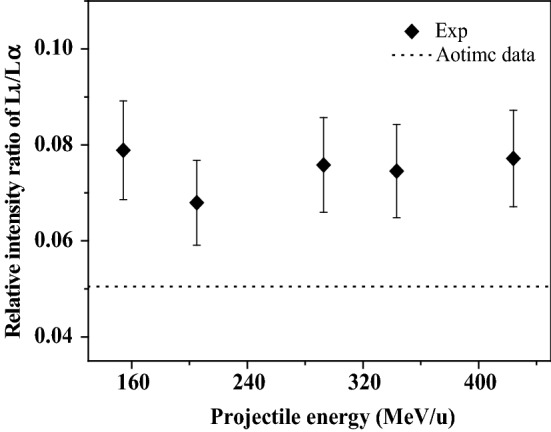
Relative intensity ratios of Au Lι–Lα x-ray induced by C^6+^ ions at different incident energy.

In addition to the blue shift of the x-ray energy, another effect of multiple ionization is to change the atomic parameters. With the presence of multiple vacancies in the outer-shells, some non-radiation transition processes, such as Auger transition and Coster-Kronig, are suppressed due to the absence of multiple electrons in the outer shells. Correspondingly, the probability of radiation transition is enlarged, which leads to the enhancement of the x-ray emission. Such change is related to the single ionization fluorescence yield. As a result, the relative intensity ratio of sub-shell x rays is altered. Table 2 present the experimental relative intensity ratios of Au L-subshell x rays, and that are also shown in Figs. 3 and 4 as a function of incident energy. The experimental data are larger than the theoretical calculations of single ionized atoms, and are almost unchanged with the increase of incident energy. This result indicates that the M and N shells of Au are multiply ionized by the fast C^6+^ ions impact, and such multi-ionization degree is almost independent of the incident energy.

**Table 2:** Relative intensity ratio of Au L-subshell x ray induced by high energy C^6+^ ions.

	Incident energy of C^6+^ ions (MeV/u)					Atomic data
	154.3	205.0	292.7	343.3	423.9	
Lβ–Lα	0.800 ± 0.112	0.768 ± 0.108	0.777 ± 0.109	0.775 ± 0.109	0.747 ± 0.105	0.580
Lι–Lα	0.079 ± 0.011	0.068 ± 0.010	0.076 ± 0.011	0.075 ± 0.010	0.077 ± 0.011	0.051

To facilitate discussion, one can divide Lβ x ray into two sets of lines. One is the Lβ_1_ and Lβ_3, 4_ × rays which have a different lower energy level with Lα. The other is Lβ_2, 15_, Lβ_5_ and Lβ_6_ × rays which have the same lower energy level with Lα. Lβ_1_ and Lα x rays are mainly the results of the radiation transition of the same upper level M_4, 5_ electrons filling the L_2_ and L_3_ vacancies respectively, and the corresponding fluorescence yields are 0.08 (ω_Lβ1_) and 0.13 (ω_Lα_) for Au. The Auger yield of L_2_ and L_3_ sub-shells are 0.544 (a_2_) and 0.680 (a_3_)^46,47^. These are all in the same scale and there is not much difference. When multiple ionization occurs in the M and N shells, a_2_ and a_3_ will be reduced by the same amplitude, which will cause the increase of ω_Lβ1_ and ω_Lα_ to be almost the same magnitude. This will not leads to a significant change in the relative intensity ratio of Lβ and Lα x rays.

However, there is an additional channel for the decay of L_2_ vacancies than that of L_3_, L_2_-L_3_X CK transition. Due to the absence of electrons in the outer shells caused by multiple ionization, part of the L_2_–L_3_X CK process is inhibited, and the fluorescence yield ω_Lβ1_ is increased. This will result in an enhancement of the Lβ_1_ x-ray emission. Similarly, the Lβ_3, 4_ x-ray emissions, the radiation transition from the decay of L_1_ shell, will also be enhanced owing to the outer-shell multiple ionization, because the probabilities of non-radiation transition filling the L_1_ vacancies are diminished by the multiple ionization. As a whole result, the relative intensity ratios of Lβ_1, 3, 4_–Lα_1, 2_ x-ray increases due to the multiple ionization of M and N shells.

Lβ_2, 15_ and Lα x rays are mainly the radiation transitions from the filling of the same lower energy level L_3_ vacancies with N_4, 5_ and M_4, 5_ electrons, respectively. When the M and N shells are multiply ionized, the Auger transition filling the L_3_ vacancies is weakened, and the corresponding process of radiation transition is enhanced. The Auger yield a_3_ of Au is 1 to 2 orders of magnitude larger than the fluorescence yield of L_3_-subshell x ray, which will increase significantly due to the decrease of a_3_, and the corresponding x-ray emission will be dramatically enhanced. The fluorescence yield of Lα x ray (ω_Lα_) is about five times as large as that of Lβ_2, 15_ (ω_Lβ1, 15_)^46,47^. ω_Lβ2, 15_ is more susceptible to the multiple ionization. Therefore, the increase of ω_Lβ2, 15_ caused by multiple ionization is greater than that of ω_Lα_. This results in a greater enhancement for L Lβ_2, 15_ × ray emissions than for Lα x ray. In the same way, the fluorescence yield of Lβ_5_ (ω_Lβ5_) and Lβ_6_ (ω_Lβ6_) are all about 2 orders of magnitude smaller than ω_Lα_. Under the multiple ionization, the increase of ω_Lβ5_ and ω_Lβ6_ is more significant than ω_Lα_. This will cause the increase of the relative intensity ration of Lβ_5, 6_ to Lα x ray.

In summary, due to the multiple ionization, the fluorescence yield Lβ x-ray present a larger enhancement than that of Lα. As a result, the Lβ x-ray emission is enhanced than that of Lα, as shown in Fig. 2. And the relative intensity ratios of Lβ–Lα x ray are higher than the atomic data, as shown in Fig. 3. But it is no obvious change with increasing incident energy, because the multi-ionization degree is almost a constant with the incident energy.

In the same way, the ratio of I(Lι)/I(Lα_1, 2_) can be understood easily. Lι and Lα x rays can be regarded as radiation transitions of different M subshell electrons filling L_3_ vacancies. The increase of fluorescence yield of those two x ray is only affected by the change in Auger yield a_3_. The fluorescence yield of Lι x ray ω_Lι_ is much smaller than ω_Lα_, which is only 5%^46,47^. Under the influence of multiple ionization, ω_Lι_ has a greater increase than ω_Lα_. As a result, the emission enhancement of Lι x ray is greater than that of Lα. As shown in Fig. 4, the relative intensity of Lι to Lα x ray is enlarged compared to the atomic data.

4. Au L-subshell x-ray production cross section induced by C^6+^ ions

In the present work, the energy loss of the projectile in the target with an extent of x-ray self-attenuation length does not exceed 0.081 MeV/u, which is much less than the initial incident energy and can be neglected. Therefore, it is proposed that the observed x rays are produced by the swift C^6+^ ions with the same energy, even though they result from the different atomic layers. Taking into account of the self-absorption of the target and the absorption of air between the target and the detector, the experimental x-ray production cross section can be extracted from the thin target formula and can be expressed as^27,48^:

1 $$\sigma_{x} = \frac{{\sqrt 2 \mu N_{x} }}{{\rho N_{p} \varepsilon_{d} f_{t} \left( {\Omega /4\pi } \right)}} \cdot \frac{1}{{1 - e^{ - \sqrt 2 \mu L} }}$$

where *μ* is the absorption coefficient of the x ray. *N*_*X*_ is the obtained counts of the x ray which were extracted by fitting the x-ray spectrum with Gauss function of origin software, namely, the area under peak of the fitted spectral line. *ρ* is the target atomic number in unit volume. *N*_*p*_ is the number of the incident ions, *ε*_*d*_ is the detector efficiency. *f*_*t*_ is the attenuation factor of the x ray in the air between the target and the detector. Ω is the solid angle. *L* is the target thickness. The principal errors for the experimental data result from the absorption of air and target 10%, x ray count statistics 5%, incident ions recording 10%, detector efficiency 5% and solid angle 6%, and the maximum error of the total cross section is about 17%.

**Figure 5:**
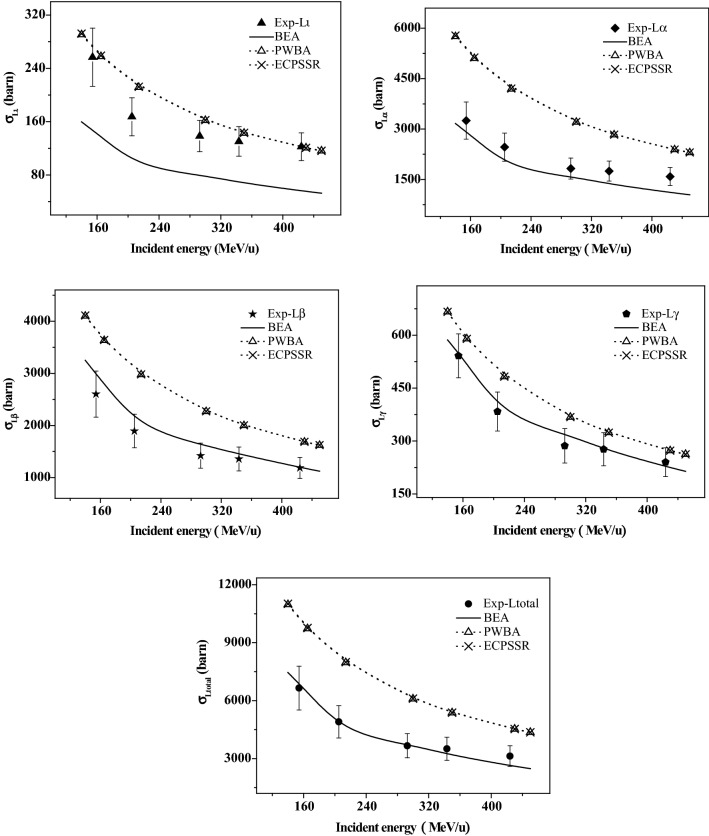
L x-ray production cross section of Au produced by high energy C^6+^ions, and compared with various theoretical calculations.

The experimental results of the L-subshell and total x-ray production cross section are listed in Table 3 as a function of the impact energy of the C^6+^ ions, and are also shown in Fig. 5. This is decreased with increasing impact energy, but the reduction is not large and only changes in the same order of magnitude, while the drop for the energy from 154.3 to 205.0 MeV/u is bigger than that in the energy region of 205.0–423.9 MeV/u. For example, for Lι x ray, the production cross section is about 10^2^ barn, which is reduced by about 53% over the entire experimental energy range, while this reduction is about 35% for the energy decreasing from 154.3 to 205.0 MeV, and 18% for 205.0–423.9 MeV/u. Furthermore, the homologous values for Lα x ray are 10^3^barn, 51, 24, 27% respectively, and 10^3^ barn, 55, 28, 27% for Lβ x ray, and 10^2^ barn, 56, 30, 26% for Lγ x ray, and 10^3^ barn, 53, 26, 27% for the total x ray.

**Table 3:** Au L-subshell x-ray production cross section induced by high energy C^6+^ ions.

E (MeV/u)	Lι (10^2^ barn)	Lα (10^3^ barn)	Lβ (10^3^ barn)	Lγ (10^2^ barn)	Ltotal (10^3^ barn)
154.3	2.57 ± 0.44	3.25 ± 0.55	2.60E ± 0.44	5.42E ± 0.62	6.65E ± 1.13
205.0	1.67 ± 0.28	2.46 ± 0.42	1.89E ± 0.32	3.84E ± 0.55	4.91E ± 0.83
292.7	1.38 ± 0.24	1.83 ± 0.31	1.42E ± 0.24	2.87E ± 0.49	3.67E ± 0.62
343.3	1.30 ± 0.22	1.75 ± 0.29	1.36E ± 0.23	2.77E ± 0.47	3.51E ± 0.60
423.9	1.22 ± 0.21	1.59 ± 0.27	1.18E ± 0.20	2.40E ± 0.41	3.13E ± 0.53

The theoretical L-subshell x-ray production cross section, σ_Lx_, can be converted from the Li-subshell ionization cross section and is calculated by the following expressions^49,50^:

2 $$\sigma_{L\iota } = \left[ {\sigma_{L1} \left( {f_{13} + f_{12} f_{23} } \right) + \sigma_{L2} f_{23} + \sigma_{L3} } \right]w_{3} F_{3\iota }$$

3 $$\sigma_{L\alpha } = \left[ {\sigma_{L1} \left( {f_{13} + f_{12} f_{23} } \right) + \sigma_{L1} f_{23} + \sigma_{L1} } \right]\omega_{1} F_{3\alpha }$$

4 $$\sigma_{L\beta } = \sigma_{L1} \omega_{1} F_{1\beta } + \left( {f_{12} \sigma_{L1} + \sigma_{L1} } \right)\omega_{2} F_{2\beta } + \left[ {\sigma_{L1} \left( {f_{13} + f_{12} f_{23} } \right) + \sigma_{L2} f_{23} + \sigma_{L3} } \right]\omega_{3} F_{3\beta }$$

5 $$\sigma_{L\gamma } = \sigma_{L1} \omega_{1} F_{1\gamma } + \left( {f_{12} \sigma_{L1} + \sigma_{L2} } \right)\omega_{2} F_{2\gamma }$$

6 $$\sigma_{LTot} = \left[ {\omega_{1} + \omega_{2} f_{12} + \omega_{3} \left( {f_{13} + f_{12} f_{23} + f_{13}^{\prime } } \right)} \right]s_{L1} + \left( {\omega_{2} + f_{23} \omega_{3} } \right)\sigma_{L2} + \omega_{3} \sigma_{L3}$$

where *σ*_*Li*_ (*i* = 1, 2, 3) is the ionization cross section of the sub-shell L_1_, L_2_ and L_3_, respectively. *f*_*ij*_ is the Coster-Kronig yield for subshells *i* to *j*. _ω*i*_ is the corresponding *i*-subshell fluorescence yield. *F*_*ix*_ is the fraction of the radiation width of the subshell L_*i*_ contained in the *x-*th spectral line.

As discussed in section 2 and 3 of “Results and discussion”, the Au atoms are multiply ionization by the fast C^6+^ ions, which result in the change of x-ray fluorescence yield and probability of no-radiation transition. The theoretical calculation of *σ*_*Lx*_ should use the parameters of multi-ionization atom. According to the research by Lapicki et al.^51,52^, the multi-ionization fluorescence yield and the CK yield are calculated in the present energy region. In the present calculations, the single ionization atomic values of *ω*_*i*_ and *f*_*ij*_ are taken from the work of Campbell^46,47^, while the data of *F*_*ix*_ is from the tables of Scofield^53,54^.

Figure 5 presents the theoretical predictions by the BEA, PWBA and ECPSSR models transformed to the x-ray production cross section, using the multiple ionization atomic parameters. They are all within the same order of magnitude and dwindle with increasing energy. The calculations of BEA are lower than that of PWBA and ECPSSR, which are almost identical, with the largest difference not exceeding 7%. ECPSSR is an improvement over the PWBA. The related corrections are significant in the lower energy region, while this can be neglected in such high energy region in this work.

The experimental-theoretical comparisons are also given in Fig. 5. As a whole, the BEA calculations are in good agreement with the experimental data in spite of the individual small deviation, except for Lι x ray, where the PWBA and ECPSSR give better estimation of the measured results. For the deviation of Lι, it is proposed that this may be caused by a larger extent anisotropic of Lι emission. In addition, the BEA simulations not only agree basically with the experiment in numerical value, but also present the best prediction of the changing trend, which dwindles faster for the incident energy dropping from 154.3 to 205.0 MeV/u than that from 205.0 to 423.9 MeV/u.

## Conclusions

The L-shell x-ray spectra of Au have been measured by bombardment of 154.3–429.3 MeV/u C^6+^ ions. The energy shift and the relative intensity ratio of the L-subshell x rays were investigated as a function of the incident energy, and the experimental x-ray production cross sections were obtained by a well corrected thick target formula and compared with the theoretical estimations of BEA, PWBA and ECPSSR. It is indicated that the Au atom is ionized mainly by direct Coulomb interaction, when it is impinged by high energy C^6+^ ions, which are moving with velocity higher than the orbital velocity of the electrons in the target atom. With the L-shell ionization, the outer-shells are multiply ionized. The multi-ionization degree is considered to be almost constant in the present energy region. This results in a significant blue shift of the x-ray energy and an enhancement of the relative intensity ratios of Lι and Lβ to Lα x ray. It is proposed that the inner-shell ionization of Au should be considered as a binary process between the high energy C^6+^ ions acting as a point charge and the independent target electrons. The L-shell x-ray production cross section can be estimated by the BEA model with the multiple ionization atomic parameters.

## Data Availability

All data generated or analyzed during this study are included in this published article.
